# Abdominal Obesity Phenotypes and Incidence of Type 2 DM, Cardiovascular Outcomes and All‐Cause Mortality—A Systematic Review and Meta‐Analysis

**DOI:** 10.1002/hsr2.72028

**Published:** 2026-05-03

**Authors:** Soraya Doustmohamadian, Marjan Momeni, Majid Mirmohammadkhani, Azam Doustmohammadian, Farhad Hosseinpanah

**Affiliations:** ^1^ Clinical Research Development Unit, Kowsar Educational, Research and Therapeutic Hospital, School of Medical Sciences Semnan University of Medical Sciences Semnan Iran; ^2^ Department of Epidemiology and Biostatistics, School of Medicine Semnan University of Medical Sciences Semnan Iran; ^3^ School of Rehabilitation Semnan University of Medical Sciences Semnan Iran; ^4^ Gastrointestinal and Liver Diseases Research Center Iran University of Medical Sciences Tehran Iran; ^5^ Obesity Research Center, Research Institute for Endocrine Sciences Shahid Beheshti University of Medical Sciences Tehran Iran

**Keywords:** abdominal obesity, cardiovascular disease, metabolically healthy obesity, mortality, type 2 diabetes mellitus

## Abstract

**Background and Aims:**

The global rise in obesity, a major driver of metabolic diseases, has prompted scrutiny of distinct obesity phenotypes. While overall obesity is concerning, abdominal obesity demonstrates a stronger association with metabolic dysfunction, type 2 diabetes (DM2), and cardiovascular disease (CVD). This review examines the risk of DM2, CVD, and mortality in adults with a metabolically healthy abdominal obese phenotype.

**Methods:**

A systematic search of PubMed/MEDLINE, Web of Science, Cochrane Library, and ProQuest was conducted on April 7, 2025, to identify prospective cohort studies in adults. Eligible studies compared MHAO individuals to metabolically healthy, non‐abdominally obese (MHNAO) controls, focusing on outcomes including incident T2DM, fatal and non‐fatal CVD events, and all‐cause mortality. Pooled estimates were calculated using random‐effects meta‐analysis, and heterogeneity was assessed using the *I*² statistic.

**Results:**

Six prospective cohort studies (*n* = 98,329) were included. Metabolically unhealthy individuals, regardless of abdominal obesity status, had significantly increased risks of T2DM (RR 9.00, 95% CI 7.51–10.50 for MUHAO; RR 5.03, 95% CI 4.11–5.94 for MUHNAO), CVD, and all‐cause mortality (HR 1.67, 95% CI 1.42–1.93 for MUHAO; HR 1.58, 95% CI 1.36–1.79 for MUHNAO). In contrast, MHAO individuals did not show significantly elevated risks of T2DM (RR 2.44, 95% CI 0.95–3.94), CVD, or all‐cause mortality (HR 1.07, 95% CI 0.88–1.27) compared to MHNAO controls. Substantial heterogeneity (*I*² > 50%) was observed, partly explained by differences in outcome definitions and metabolic classifications.

**Conclusion:**

While metabolically unhealthy phenotypes are strongly associated with adverse health outcomes, individuals with MHAO appear to have risk profiles comparable to their metabolically healthy, non‐abdominally obese counterparts. Nevertheless, abdominal adiposity and metabolic status remain critical determinants of long‐term health, and the MHAO phenotype may not be entirely benign.

**Trial Registration:** PROSPERO (CRD42019111056)

## 1 Introduction

The global prevalence of obesity has reached alarming levels, affecting more than 2 billion adults worldwide and posing a major public health challenge across high‐, middle‐, and low‐income countries alike [1]. It is linked to severe health consequences such as insulin resistance, dyslipidemia, diabetes mellitus type 2 (DM2), coronary artery events, and hypertension [2]. However, it has been observed that using body mass index (BMI) as a sole indicator of obesity may not provide a comprehensive definition. Several studies have demonstrated that BMI‐defined obesity is insufficient to fully capture the definition of obesity [3, 4].

Various forms of obesity result from the interaction of metabolic factors and obesity, each with potentially various risks for later health conditions, including cardiovascular disease, DMT2, and all‐cause mortality. A subgroup of obese people with a BMI higher than 30 kg/m² but without metabolic abnormalities is considered metabolically healthy obesity (MHO) [5, 6]. The prevalence of MHO varies between 10% and 40%, depending on the benchmarks used to define metabolic health in studies. It has been suggested that MHO may not increase the risk of cardiovascular disease or all‐cause mortality [7, 8]. However, some data have shown an increased risk of cardiovascular disease and mortality in individuals classified as MHO when compared to those with normal weight and a metabolically healthy phenotype (MHNW) [9, 10].

The limitations of using BMI alone in capturing cardiometabolic risk can be linked to the issue that body mass index does not adequately account for total body fat, particularly intraabdominal visceral fat [11]. It is well‐established that waist circumference (WC) varies considerably within a given BMI category, and individuals with higher WC values within the same BMI category are at a higher risk of adverse health effects [12, 13, 14]. Compared to BMI, WC predicts health outcomes more confidently, potentially due to its ability to identify individuals with higher visceral adipose tissue (VAT) mass. Abdominal obesity is more highly associated with metabolic dysfunction than generalized obesity [15, 16]. As revealed by many studies, dyslipidemia, DM2, coronary artery events, and hypertension are all independently associated with abdominal obesity as a risk factor. Research shows an increase in cardiovascular death and all‐cause mortality associated with higher values of waist circumference in abdominal obese populations [17, 18, 19]. Additionally, WC was identified as a critical factor for measuring the reduction in cardiovascular disease risk following the adoption of healthy behaviors [20].

The interaction between metabolic risk factors and waist circumference can be observed in abdominal obesity phenotypes [21]. It has been suggested that abdominal obesity, particularly waist circumference, is a better indicator for predicting health outcomes [15, 22]. A small cohort of individuals with central obesity but with no metabolic disorders attributed to obesity was diagnosed as having metabolically healthy abdominal obesity (MHAO). MHAO refers to individuals with abdominal obesity without dyslipidemia, insulin resistance, or hypertension [23]. The literature revealed that approximately 23.5% of individuals with abdominal obesity can be classified as having MHAO [10, 24].

This meta‐analysis is the first to systematically evaluate the MHAO phenotype, offering critical insights into its prognostic value for T2DM, CVD, and mortality, with implications for refining obesity risk stratification.

## 2 Methods

The study commenced with the formulation and publication of the protocol on the PROSPERO website. Subsequently, upon receiving the protocol code from PROSPERO (CRD42019111056), the systematic search was conducted in the following manner:

### 2.1 Data Sources and Search Strategies

A comprehensive systematic search was performed in Scopus, Web of Science, MEDLINE (PubMed), the Cochrane Library, and ProQuest to identify studies investigating abdominal obesity phenotypes and their associated outcomes, specifically DM2, all‐cause mortality, and cardiovascular disease. Articles were retrieved using specific keywords such as “central adiposity,” “abdominal obesity*,” “Cardiovascular Diseases,” “Obesity, Abdominal,” “Type 2 Diabetes Mellitus,” “obesity phenotype”, “mortalit*,” “abdominal fat*,” and “metabolically healthy.” These keywords were enclosed in quotation marks, and wildcards (*) were employed. Boolean operators ‘AND’ and ‘OR’ combined the keywords. A subject search was also conducted using Medical Subject Headings (MeSH) through the Medline (PubMed) database. To maximize the comprehensiveness of the search, cited articles were examined through forward searching, reference lists of meta‐analyses and review papers were screened through backward searching, and “related articles” suggested by indexing databases were also reviewed. The search and data screening/selection strategies pursued the Preferred Reporting Items for Systematic Reviews and Meta‐analyses (PRISMA) guidelines for this study.

### 2.2 Selection of Studies and Eligibility Criteria

The review study assessed the consequences of phenotypes related to abdominal obesity without restricting the types of studies included. Consequently, all original studies published in scientific journals until April 7, 2025, were incorporated into this study without imposing any time constraints. The search was conducted exclusively in English, and only studies involving human subjects aged 18 years and above were considered. This age restriction was applied because the metabolic and hormonal determinants of abdominal obesity differ substantially between adults and younger populations. Including children and adolescents could introduce clinical heterogeneity, and pediatric obesity has been comprehensively addressed in separate systematic reviews.

**Figure 1:**
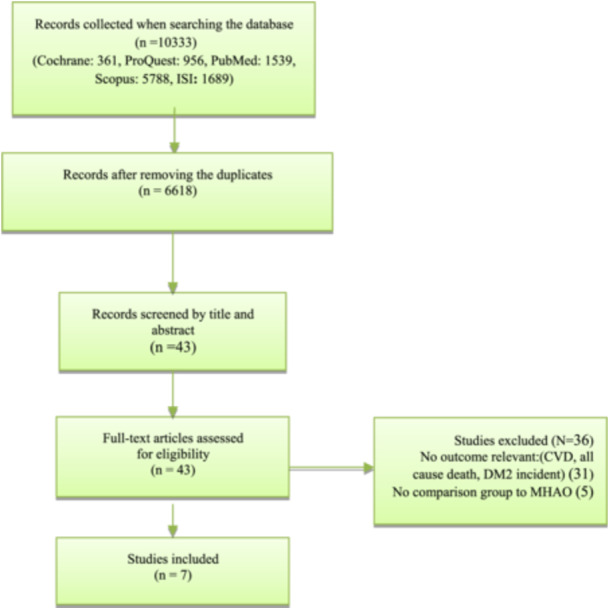
The flowchart depicting the process of paper search and selection based on the PRISMA.

The study encompassed all cohort studies (both prospective and retrospective) among adults that defined a metabolically healthy abdominal obese group and examined the correlation between different phenotypes of people with abdominal obesity and the incidence of type 2 diabetes mellitus, cardiovascular events (fatal or non‐fatal), and all‐cause mortality. Studies that did not include a comparison group for the Metabolic Healthy Abdominal Obese (MHAO), or were based on a combination of BMI (obesity) and metabolic index (MHO phenotype), did not have accessible abstract and full text, or had examined outcomes other than DM2, cardiovascular events, or all‐cause death were excluded (Figure 1).

The initial stage involved searching for and retrieving articles. Subsequently, after removing duplicate articles, two project collaborators evaluated their titles and abstracts independently to identify the eligible articles and exclude any unrelated ones. Two reviewers (Soraya Doustmohamadian and Marjan Momeni) criticized the selected articles independently to enhance their quality and mitigate potential biases. In disagreements, consultations with an expert investigator were conducted to resolve the discrepancies.

### 2.3 Study Quality Assessment

Two project collaborators who independently assessed the eligible articles evaluated the study quality. The researchers, Soraya Doustmohamadian and Marjan Momeni, also independently evaluated the quality of the papers using the Newcastle–Ottawa scale (NOS) and the Quality Assessment Form for the final sample of the retrieved studies. The classification of the studies was conducted according to the retrieved article selection, degree of group comparability, and evaluation of the results, with categorizations into three levels of quality: good, fair, and poor. For considering an article as good quality, these conditions were the minimum: the selection domain with 3 or 4 stars, the comparability domain with 1 or 2 stars, and the outcome domain with 2 or 3 stars. The criteria for determining fair quality were as follows: in the selection domain, two stars; in the comparability domain, one or two stars; and in the outcome/exposure domain, two or three stars. The criteria for determining poor quality were a score of 0 or 1 in the comparability, outcome/exposure, or selection domains. Only articles achieving a quality score of six or higher were included in the analysis. The strengths and weaknesses of each article were assessed and documented. To minimize bias, a comprehensive search was conducted in various international databases.

### 2.4 Data Extraction

An extraction form was created to extract the data from the articles. This form included details on study characteristics (such as year of publication, the title of the journal, design, and the time of intervention), characteristics of the participants (such as gender, age, sample size, and the way abdominal obesity and metabolic syndrome were defined), and the outcomes of interest (i.e., DM2, risk of all‐cause mortality, and/or cardiovascular disease were documented).

### 2.5 Data Analysis

The data were analyzed using Stata 11.2, according to the “metan” command. The I‐squared index was utilized to verify the heterogeneity, with values above 50 indicating heterogeneity. This meta‐analysis employed the random‐effects method, and a forest plot was generated for each analysis. Meta‐regression was utilized to investigate potential sources of heterogeneity for the total estimate.

## 3 Results

Adopting the PRISMA flowchart diagram, as displayed in Figure 1, the process of article selection and the related criteria for the inclusion of the articles in the present study were conducted.

### 3.1 Eligible Studies

The results of the article selection process, along with the related criteria, are presented in Figure 1, which illustrates the number of articles included in the systematic review following the initial search. An initial search yielded 10,333 articles (1539 from PubMed, 1686 from Web of Science, 361 from the Cochrane Library, and 5788 from Scopus), and 6618 unique records remained after deduplication. Following rigorous screening, seven articles met the eligibility criteria and were included in this study. These papers, published between 2012 and 2023, formed the basis of the analysis. A total of seven prospective cohort studies were identified that examined the MHAO phenotype and its association with cardiovascular outcomes, all‐cause mortality, or incident diabetes. The specific details of these studies are presented in Tables 1 and 2.

**Table 1:** Characteristics of included studies.

Study, year (reference)	Sample size	Baseline CVD	Age, years men, %	Median follow‐up, years	Primary outcome	NOS, point
Hamer et al. 2012 [25]	22,203	No	54 years, 45.2%	7	CVD mortality and all‐cause mortality	Good quality, 6
Daphne L. van der A et al. 2014 [26]	22,654	Yes	20–59 years, NA	13.4	All‐cause mortality	Good quality,7
Keihani et al. 2015 [24]	7122	No	47.4 (12.4), 42.7%	10	CVD	Good quality, 6
Heianza et al. 2014 [9]	29,564	Yes	50.7 (8.7) 64%	5	DM2 incident	Good quality, 6
Doustmohamadian et al. 2017 [10]	8804	Yes	47.7 (12.6), 45.2%	12.5	All‐cause mortality	Good quality,7
Salehinia et al. 2018 [27]	7982	Yes	42.2 ± 14.5, 44%	12.0	DM2 incident	Good quality,7
Xiaoyan Wu et al. 2023 [28]	15,704		51 years, 38.86%	6	All‐cause mortality	Good quality,7

Abbreviations: CVD = cardiovascular disease, DM = diabetes mellitus, NA = not available, NOS = Newcastle–Ottawa scale.

The average follow‐up period for the four studies was approximately 12 years. One cohort study investigated the interplay between the MHAO phenotype and cardiovascular disease risk [24], while three examined all‐cause mortality in people with the MHAO phenotype [10, 26, 28]. Additionally, two cohort studies investigated the incidence of type 2 diabetes mellitus concerning the MHAO phenotype [9, 27]. In one study, a separate analysis was conducted to examine the outcomes of CVD mortality and all‐cause mortality in individuals with the abdominal obesity phenotype, in addition to BMI, with a follow‐up period of 7 years [25].

### 3.2 Definitions of MHAO

In two studies, abdominal obesity was defined as having a waist circumference (WC) greater than 89 cm for men and greater than 91 cm for women (Table 2) [10, 24]. In one study, the national cutoff point for WC (90 cm) was adopted to define abdominal obesity in both men and women [29]. In two other studies, abdominal obesity was defined as a waist circumference of 102 cm or higher for men and 88 cm or higher for women [25, 26]. In another study, abdominal obesity was defined as having a waist circumference of 90 cm or higher for men and 80 cm or higher for women [9, 28]. In four studies, metabolic health was defined as having less than or equal to one component of metabolic syndrome (excluding WC) according to the Joint Interim Statement (JIS) definition [10, 24, 28, 30].

**Table 2:** MHAO definition of included studies.

Study, year (reference)	Definition of metabolically healthy	Definition of abdominal obesity
Hamer et al. 2012 [25]	≤ 1 metabolic abnormality based on BP, DM, WC, HDL, CRP > 3	Based on WC > 102 cm in men and > 88 cm in women
Daphne L. van der A et al. 2014 [26]	No metabolic risk factors based on ATP III	Based on WC > 102 cm in men and > 88 cm in women
Sorena Keihani et al. 2015 [24]	As ≤ 1 components of metabolic syndrome (excluding WC), using the JIS definition.	WC cutoff points of > 89 cm for men and > 91 cm for women
Heianza et al. 2014 [9]	< 1 metabolic abnormality based on BP, DM, TG, HDL	WC cutoff points of > 90 cm for men and > 80 cm for women
Doustmohamadian et al. 2017 [10]	As ≤ 1 components of metabolic syndrome (excluding WC), using the JIS definition	WC cutoff points of > 89 cm for men and > 91 cm for women
Salehinia et al. 2018 [27]	≤ 1 metabolic abnormality based on JIS	WC > 90 cm for men/women
Xiaoyan Wu et al. 2023 [28]	No metabolic risk factors based on JIS	Based on WC > 90 cm in men and > 80 cm in women

Abbreviations: ATP = adult treatment panel, BP = blood pressure, CRP = C‐reactive protein, CVD = cardiovascular disease, DM = diabetes mellitus, HDL= high‐density lipoprotein‐cholesterol, JIS = joint interim statement, MHAO = metabolically healthy abdominal obese, WC = waist circumference.

In one study, not having any metabolic risk factors from the Adult Treatment Panel (ATP) III criteria for metabolic syndrome [12] was considered “metabolically healthy” [26]. In the Hammer study, metabolic health was defined as having less than or equal to one component of metabolic abnormalities based on blood pressure, high‐density lipoprotein‐cholesterol (HDL), diabetes mellitus diagnosis, and C‐reactive protein (CRP) being greater than 3 mg/L [25]. Another study defined metabolic health as having no metabolic factors based on hypertension, hypertriglyceridemia, low HDL, and impaired fasting glucose (IFG) [9].

### 3.3 Outcomes Measured

Four studies reported all‐cause mortality [10, 25, 26, 28], with all four studies using metabolically healthy, non‐abdominal obese (MHNAO) as the comparison group. In the EPIC study, separate categorization was performed based on waist‐defined obesity, and “metabolically healthy” normal waist (MHNW) was ranked as the reference group (Table 2). Two studies reported cardiovascular disease (CVD) as the primary outcome [24, 25]. CVD was defined as any cardiac heart disease (CHD), which included cases of definite or probable myocardial infarction and proven CHD by angiography, stroke (a new neurological deficit that lasted more than 24 h), or CVD death. In the Hammer study, a second set of analyses was done based on CVD mortality (no cardiovascular event). Two studies reported DM2 as the primary outcome [9, 30]. According to the American Diabetes Association (ADA) criteria at any phase of the follow‐up, DM2 was defined as fasting plasma glucose (FPG) higher than or equal to 126 mg/dL (7.0 mmol/L) or 2‐h post‐load plasma glucose (2 h‐PLPG) higher than 200 mg/dL (11.1 mmol/L) or being treated with anti‐diabetic medicine [31].

In this meta‐analysis, the consequences of cardiovascular events and cardiac or all‐cause mortality were evaluated collectively. A composite endpoint comprising cardiovascular events, cardiac death, or all‐cause death was constructed.

### 3.4 Abdominal Obesity Categories and DM2

**Figure 2:**
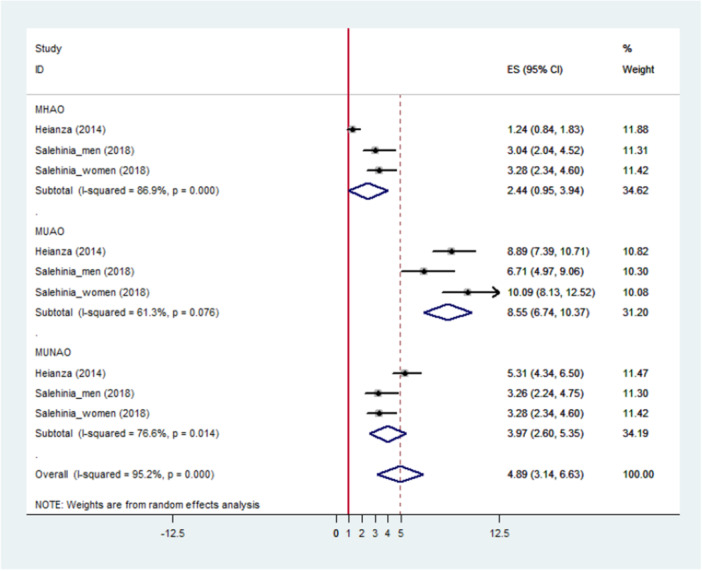
Meta‐analyses of metabolically healthy waist circumference categories for the risk of type 2 DM compared with metabolically healthy non‐abdominal obese persons.

In a pooled analysis of two studies, Figure 2 demonstrates that both metabolically unhealthy participants with abdominal obesity and non‐abdominal obese metabolically unhealthy participants are at a higher risk of DM2 compared to their counterparts (RR, 9.00; CI, 7.51–10.50 and RR, 5.03; CI, 4.11–5.94). Individuals with MHAO were not at a higher risk for DM2 (RR, 2.44; CI, 0.95–3.94). There was significant heterogeneity in both total and individual estimates (I‐squared > 50%; *p* < 0.05), except for individuals with MUAO (*p* = 0.076).

### 3.5 Abdominal Obesity Categories and CVD/CV or All‐Cause Mortality

**Figure 3:**
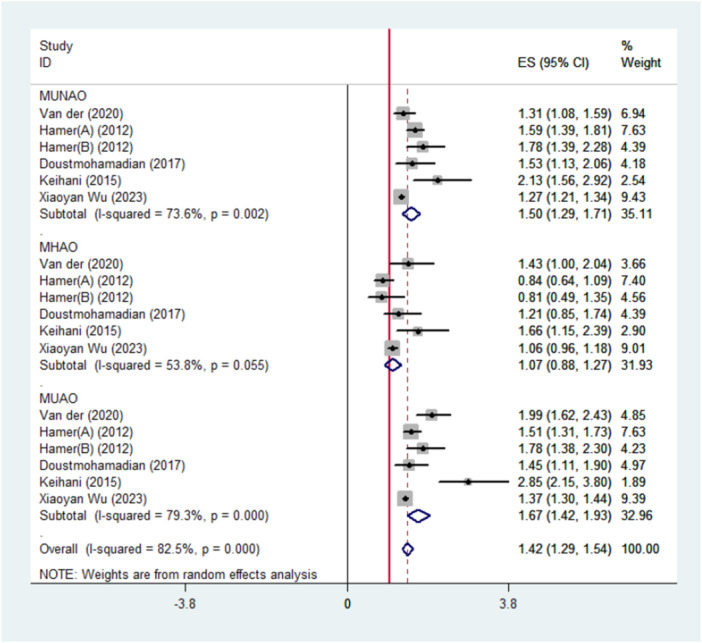
Meta‐analyses of metabolically healthy waist circumference categories for the risk of all‐cause mortality and/or cardiovascular events compared with metabolically healthy non‐abdominal obese persons (reference).

In six pooled analyses, it was revealed that both the group with metabolically unhealthy abdominal obesity (MUHAO) and the group without abdominal obesity (MUHNAO) had a greater risk for cardiovascular events or all‐cause mortality in comparison with the group with metabolically healthy non‐abdominal obesity (MHNAO) (HR, 1.67; CI, 1.42–1.93 for MUHAO and HR, 1.58; CI, 1.36–1.79 for MUHNAO) (Figure 3). Figure 3 also indicates that individuals with metabolically healthy abdominal obesity (MHAO) were not at a higher risk for all‐cause mortality or cardiovascular events (HR, 1.07; CI, 0.88–1.27).

A significant heterogeneity was found in the total estimates (I‐squared = 82.5%; *p* < 0.05). The heterogeneity in all subgroups was also greater than 50% (*p *< 0.05). As shown in Figure 5, the total and subgroup heterogeneity decreased after excluding the study by Keihani et al., which focused solely on cardiovascular disease as the primary outcome. However, it decreased for individuals with MUHNAO (I‐squared = 70.3%; *p* = 0.139) and is no longer significant for individuals with MUHAO. Meta‐regression analyses revealed that the source of heterogeneity in the total estimate could be partly attributed to the outcome of interest (cardiovascular disease vs. all‐cause mortality; *p* = 0.043) as well as the definitions of the metabolic categories (MUHNAO, MHAO, and MUHAO; *p* = 0.021).

### 3.6 Clinical Characteristics Based on Abdominal Obesity Category and Metabolic Status

**Figure 4:**
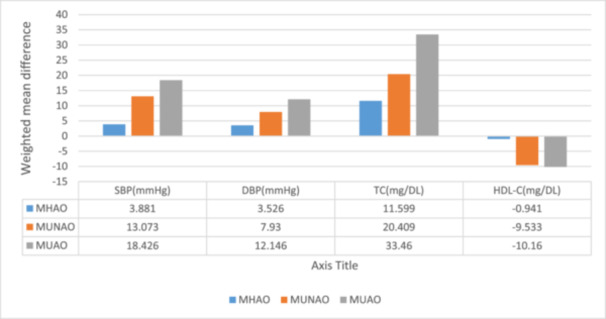
Meta‐analyses of various clinical characteristics by metabolic categories in contrast with MHNAO.

Baseline clinical characteristics were subsequently compared across abdominal obesity categories, stratified by metabolic status, using data from studies that reported the relevant information. Figure 4 displays the weighted mean difference of each clinical characteristic compared to the group with metabolically healthy non‐abdominal obesity. The metabolically healthy and unhealthy strata observed increased systolic and diastolic blood pressure, total cholesterol level, and HDL cholesterol. For each of these risk factors, those with a larger waist circumference had higher levels when compared to individuals with the same metabolic status (healthy or unhealthy), and a similar inverse interplay was seen for HDL.

**Figure 5:**
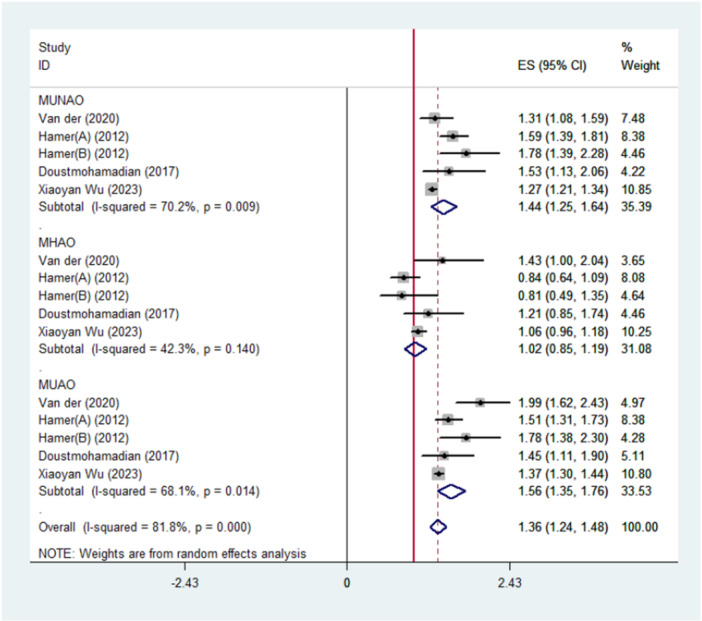
Meta‐analyses of metabolically healthy waist circumference categories for the risk of all‐cause or cardiovascular mortality compared with metabolically healthy non‐abdominal obese persons as reference (*Note:* The study by Kayhani et al. [24] was excluded from the meta‐analysis due to lack of relevant data).

## 4 Discussion

This systematic review sought to comprehensively evaluate the interplay between the MHAO phenotype and the risks of cardiovascular disease, type 2 diabetes mellitus (DM2), and all‐cause mortality. The key findings can be summarized as follows: metabolically healthy individuals with abdominal obesity do not exhibit an increased risk of CVD, DM2, or all‐cause mortality compared to metabolically healthy individuals without abdominal obesity. Second, all phenotypes characterized by an unhealthy metabolic status, regardless of abdominal obesity, demonstrate a heightened risk of DM2, mortality, and CVD. Third, as waist circumference increases, both systolic and diastolic blood pressure and total cholesterol levels also rise, while HDL cholesterol levels decline, observed in both metabolically healthy and unhealthy subgroups. Prior meta‐analyses have predominantly explored the association between BMI‐defined obesity categories and the risk of CVD, mortality, or DM2 [32, 33]. Notably, this systematic review represents the first meta‐analysis that explicitly categorizes based on abdominal obesity.

Significant variability in the definitions of MHAO across studies posed substantial challenges to quantitative synthesis. Waist circumference thresholds differed between populations and guidelines; some studies applied lower cutoffs for Asian populations compared with European cohorts, reflecting known ethnic differences in cardiometabolic risk at similar WC levels. Additionally, measurement techniques varied, with some studies following WHO protocols and others using NIH guidelines, which may yield slightly different WC values. Although the number of included studies was relatively small, this allowed for a focused assessment of differences in definitions and their potential impact on outcomes. However, the small sample limits the ability to perform subgroup analyses by ethnicity or measurement protocol.

The most prevalent definition of MHAO entails the presence of no more than one component of metabolic syndrome (excluding waist circumference), as per the guidelines established by the Japanese Society of Internal Medicine (JIS), with waist circumference measurements exceeding 89/91 cm for men/women. In contrast, other studies, such as the Epic study, employed diverse definitions: waist‐defined obesity categories combined with varying thresholds for metabolic health [26]. For example, one approach defined “metabolically healthy” as the absence of metabolic risk factors per the Adult Treatment Panel III (ATP III) criteria, while another allowed for no more than one metabolic abnormality [34]. In the Hamer study, MHAO was identified as the presence of no more than one metabolic abnormality, which encompassed blood pressure exceeding 130/85 or the use of antihypertensive drugs, suboptimal high‐density lipoprotein cholesterol levels (less than 1.03 mmol/L for men and less than 1.3 mmol/L for women), a diagnosis of diabetes mellitus, waist circumference exceeding 88 cm for men and 102 cm for women, and low‐grade inflammation (C‐reactive protein exceeding 3 mg/L) [25]. Most studies employed metabolically healthy, non‐abdominally obese (MHNAO) people as the reference group [28, 35].

While metabolically unhealthy phenotypes, irrespective of abdominal obesity, consistently conferred elevated risks of DM2, mortality, and CVD, the risk in MHAO individuals compared to MHNAO individuals was not uniformly elevated [36, 37]. Findings across the included studies were inconsistent, leaving the question of whether these variations reflect methodological differences, such as sample size and follow‐up duration, or genuinely weak associations. Notably, the length of follow‐up appeared critical. In studies with extended follow‐up periods (> 10 years) by Van Der A et al., Keihani et al., and Salehinia et al., increased risks of DM2, CVD, and mortality among MHAO individuals emerged only after approximately a decade [24, 30, 36]. As depicted in Figure 3, the random effect analyses conducted on the studies indicated that MHAO was not correlated with a higher incidence of CVD and mortality. In a cohort study in a Chinese population with a mean age of 90, total and cardiac mortality were lower in the group MHAO (waist circumference greater than 80 in women and 85 in men), and MHAO predicts better survival among the Chinese oldest [38].

Nonetheless, these studies varied regarding follow‐up duration, sample size, definitions of metabolically healthy status, abdominal obesity thresholds, and measurement techniques. These differences in definitions and protocols likely contributed to the observed heterogeneity in outcomes. Meta‐regression analyses identified outcome and exposure as sources of heterogeneity rather than follow‐up duration alone. Since all studies assessed the incidence of all‐cause mortality or CVD according to the Hazard Ratio (HR), which accounts for the effect of time, the divergent results found in these studies cannot be solely linked to differences in the length of the follow‐up period.

### 4.1 Limitation

One limitation of our findings is the considerable diversity observed for each risk. Meta‐regression analyses conducted on all groups with metabolically healthy abdominal obese (MHAO) and metabolically unhealthy non‐abdominal obese (MUHNAO), metabolically unhealthy abdominal obese (MUHAO) identified that the primary outcome and exposure utilized in each study were contributing factors to the observed diversity. Furthermore, the studies did not provide descriptions of the length of the exposure time to the current WC and metabolic factors, as well as longitudinal changes in abdominal obesity and metabolic status, which could influence the estimates. Differences in ethnic cutoffs and WC measurement methods further limit comparability across studies. Thus, it can be posited that the potential confounding effect of longitudinal changes in waist circumference and metabolic status in our analyses was likely conservative. Another significant limitation in our statistical analyses was the utilization of unadjusted pooled estimates in this meta‐analysis; therefore, we did not take into consideration other covariates that may be interplayed with CVD, mortality, and DM2, such as physical activity and, most importantly, smoking.

### 4.2 Clinical and Public‐Health Implications

The results have several implications. Clinically, reliance on a single cross‐sectional classification of metabolic health in patients with abdominal obesity may underestimate future risk; longitudinal monitoring of metabolic markers and waist circumference is warranted. From a public‐health perspective, interventions should target metabolic risk factors irrespective of BMI or waist circumference, while acknowledging that abdominal adiposity may accelerate metabolic decline. Policies that combine obesity prevention with metabolic‐risk screening could more effectively identify individuals at imminent risk.

### 4.3 Strengths and Limitations

Strengths include a comprehensive search strategy focused specifically on abdominal obesity phenotypes and the use of meta‐analytic techniques and meta‐regression to explore heterogeneity. Important limitations deserve emphasis. First, pooled estimates were derived largely from unadjusted or inconsistently adjusted data, preventing definitive control for confounders such as smoking, physical activity, socioeconomic status, and medication use. Second, most included studies lacked repeated measures of waist circumference and metabolic status, limiting inference about phenotype transitions and the temporal relationship between adiposity change and outcomes. Third, definitions of both abdominal obesity and metabolic health varied across studies, reducing comparability. Finally, language restriction to English may have excluded relevant studies.

### 4.4 Future Research Directions

Future studies should adopt longitudinal designs with repeated measures of waist circumference and metabolic biomarkers to characterize transitions between phenotypes and to estimate time‐dependent risk. Harmonization of definitions for abdominal obesity and metabolic health, ideally through consensus thresholds that account for sex and ethnicity, would reduce heterogeneity. Individual participant data (IPD) meta‐analyses could enable adjustment for key confounders and exploration of effect modification by age, sex, ethnicity, and baseline metabolic profile.

## 5 Conclusion

In conclusion, this meta‐analysis underscores the metabolic heterogeneity that exists among individuals with comparable waist circumference. Metabolically unhealthy individuals, irrespective of abdominal obesity, consistently demonstrate a significantly higher risk of all‐cause mortality, cardiovascular events, and type 2 diabetes mellitus when compared to metabolically healthy individuals without abdominal obesity. Conversely, metabolically healthy individuals with abdominal obesity do not appear to face a substantially increased long‐term risk for these outcomes. Nevertheless, both abdominal adiposity and metabolic dysfunction remain independent predictors of adverse cardiometabolic outcomes. The apparent absence of elevated risk in the MHAO phenotype may reflect a transient metabolic state rather than a truly benign condition. Shorter‐term studies may fail to capture the progressive risk associated with this phenotype. Future investigations should adopt longitudinal designs capable of tracking dynamic changes in waist circumference and metabolic health over time, offering a more nuanced understanding of risk trajectories across metabolic‐abdominal phenotypes.

## Author Contributions

**Soraya Doustmohamadian:** conceptualization, data curation, investigation, validation, writing – original draft, writing – review and editing. **Marjan Momeni:** writing – review and editing, data curation, investigation, validation. **Majid Mirmohammadkhani:** writing – review and editing, software, formal analysis, methodology, visualization, and data curation. **Azam Doustmohammadian:** writing – review and editing, writing – original draft, and validation. **Farhad Hosseinpanah:** conceptualization, validation, project administration, supervision, data curation writing – review and editing.

## Funding

The authors have nothing to report.

## Conflicts of Interest

The authors declare no conflicts of interest.

## Transparency Statement

The corresponding author, Farhad Hosseinpanah, affirms that this manuscript is an honest, accurate, and transparent account of the study being reported; that no important aspects of the study have been omitted; and that any discrepancies from the study as planned (and, if relevant, registered) have been explained.

## Data Availability

Data and materials are available within the complementary materials, and further information can be available by request to the corresponding author Farhad Hosseinpanah (farhad.hosseinpanah@gmail.com).
